# Development and Comparison of Visual Colorimetric Endpoint LAMP and Real-Time LAMP-SYBR Green I Assays for *Alternaria alternata* (Fr.) Keissl in *European Plum*

**DOI:** 10.3390/jof12010056

**Published:** 2026-01-12

**Authors:** Hongyue Li, Canpeng Fu, Pan Xie, Wenwen Gao, Zhiqiang Mu, Lingkai Xu, Qiuyan Han, Shuaishuai Sha

**Affiliations:** 1 School of Advanced Agricultural Sciences, Kashi University, Kashi 844008, China; 18738665709@163.com (H.L.); fucp000@outlook.com (C.F.); panxie126@sina.com (P.X.);; 2 Research Center for Crop Biotechnology Breeding and Smart Cultivation in Southern Xinjiang, Kashi 844000, China

**Keywords:** LAMP, *European plum* brown spot, molecular detection, Cresol Red, SYBR Green I

## Abstract

*European plum* (*Prunus domestica* L.) is widely cultivated worldwide, with China producing 6.8 million t annually (55% of the global total output). However, the Kashgar region of Xinjiang, China’s primary production area, has experienced outbreaks of brown spot disease caused by *Alternaria alternata* (Fr.) Keissl. Outbreaks of this disease severely hinder both domestic and global development of the *European plum* industry. Because this pathogen has a strong latent infection capability during the early stages of disease development, its early detection is important. We develop two detection methods targeting the ITS sequence of *A. alternata*: LAMP-Cresol Red chromogenic visible endpoint detection and LAMP-SYBR Green I real-time fluorescent detection. Both methods demonstrate high specificity for *A. alternata*, enabling stable detection of the pathogen in various plant samples; detection limits reach the femtogram (fg) level, significantly surpassing conventional PCR detection capabilities. Development of these highly efficient and precise early detection methods provides a solid foundation for sustainable development of China as a global hub of the *European plum* industry, and contributes significantly to global disease prevention, control, and industrial stability for this crop.

## 1. Introduction

The cultivated *European plum* (*Prunus domestica* L.) is native to Western Asia and Europe. This plum is now cultivated across all major continents, and widely so across Asia, Europe, Africa, the Americas, and Oceania. Food and Agriculture Organization (FAO) data for 2023 reveal 30 countries to have average yields exceeding 10,000 kg/h, of which plum production throughout Asia exceeded 8 million metric tons (8 Mt, 66% of global production), of which China produced 6.8 Mt (55% of the global production) (https://www.fao.org/faostat/zh/#data/QCL/visualize (accessed on 25 November 2025)). These data reveal China to be the world’s leading producer of *European plum*.

Owing to its sweet-sour taste and high nutritional value, *European plum* has been widely introduced and cultivated across China, primarily in Xinjiang, Shaanxi, Hebei, and Henan. In Jiashi county (Xinjiang), a leading national production area, plum cultivation covers 57,000 hectares (~855,000 mu), with an annual output of 370,000 t, and contributes to a per capita income increase of 17,000 CNY [1]. This has established this industry as a distinctive regional specialty and a vital driver of local prosperity [2]. However, *European plum* brown spot frequently erupts in major production areas such as Jiashi and Yingjisha counties, and its impact is becoming increasingly severe.

*Alternaria alternata* (Fr.) Keissl. is the dominant pathogenic agent of *European plum* brown spot in Xinjiang, China (Sha et al., unpublished). The early spots were light brown and appeared as circular or irregular lesions. Over time, these spots developed into brown to brown-black large necrotic regions covering the entire fruit surface. Eventually, the diseased fruits fell off the tree [3]. In addition, *A. alternata* is known to produce several mycotoxins, including alternariol, alternariol monomethyl ether, and altertoxins, which may pose potential food safety risks to consumers when infected fruits are consumed [4].

Current molecular detection technologies for *A. alternata* are constrained by significant limitations. On one hand, reported PCR and qPCR methodologies exhibit strong dependence on sophisticated laboratory infrastructure, thereby precluding their practical deployment in field settings. On the other hand, although loop-mediated isothermal amplification (LAMP) assays developed for *A. alternata* demonstrate potential for on-site diagnostics, their analytical sensitivity (detection limit of 1 fg) remains suboptimal and necessitates further improvement. Furthermore, detection research specifically targeting *A. alternata* on *European plum* as a defined host is presently lacking, representing a critical knowledge gap. We aim to establish a robust molecular detection method for *A. alternata* on *European Plum* to facilitate its early detection and real-time field monitoring to contain its spread and safeguard agricultural production.

Loop-mediated isothermal amplification (LAMP) [5] is increasingly used in molecular diagnostics. Compared with conventional PCR, LAMP uses a strand-displacing Bst polymerase to perform auto-cycling strand displacement DNA synthesis under isothermal conditions (60 to 65 °C for 45 to 60 min). The reaction requires four to six primers that recognize six to eight distinct regions of the target DNA sequence, and confers high amplification specificity [6]. Applications of LAMP extend across diverse fields including medicine [7,8], food engineering [9,10], and agricultural and forestry sciences [11,12]. LAMP products were initially detected using agarose gel electrophoresis, as in PCR detection, but this approach is of limited use in practical situations. While an end-point SYBR Green I fluorescence detection method was then developed [13], the need to open reaction tubes can result in aerosol contamination. Various closed-tube detection strategies (e.g., magnesium pyrophosphate turbidimetry [14,15,16], hydroxynaphthol blue (HNB) colorimetric assays [9,17,18], real-time loop-mediated isothermal amplification (LAMP) with fluorescent probes [19,20], and EvaGreen real-time fluorescence detection [21,22]) were then developed. Moreover, the principles and comparative advantages of LAMP are well articulated in several technical reviews. These resources collectively provide a comprehensive foundation for understanding the rationale behind its selection and adaptation in our study [23,24]. While these methods can directly or indirectly detect DNA amplification [23], their application in the field is also problematic.

To enhance field applicability, pH-dependent visual colorimetric detection methods [25,26,27] have been proposed. During nucleic acid amplification in a LAMP system, substantial amounts of pyrophosphate and hydrogen ions are released as byproducts, and these decrease pH by 2 to 3 units at the end of the reaction. The absence of a buffering system and the associated pH change do not significantly affect DNA polymerase activity or DNA amplification [28].

We develop a visual loop-mediated isothermal amplification system (LAMP-Cresol Red) and a SYBR Green I-integrated loop-mediated isothermal amplification system (LAMP-SYBR Green I) to concurrently detect *A. alternata*. The visual LAMP-Cresol Red system uses the pH indicator cresol red, where a distinct color shift serves as an indirect yet reliable indicator of amplification, making it particularly suitable for field applications because of its pronounced visibility. In contrast, the LAMP-SYBR Green I system uses SYBR Green I as a fluorescent intercalating dye to enable real-time fluorescence monitoring. We evaluate and compare these two systems to assess their specificity, sensitivity, robustness, and field applicability.

## 2. Materials and Methods

### 2.1. Tested Strains

To evaluate the specificity of the LAMP assay, 15 strains of *A. alternata* sourced from the College of Modern Agriculture at Kashi University were randomly selected. In addition, eight other pathogenic fungal species were included for specificity testing, comprising fungi commonly associated with *European plum* (*Aspergillus ochraceus*, *Chaetomium globosum*, and *Neoscytalidium dimidiatum*) and pathogens prevalent on fruit trees in the Xinjiang region (*Diaporthe phaseolorum*, *Cytospora chrysosperma*, *Botryosphaeria dothidea*, *Fusarium oxysporum*, and *Fusarium verticillioides*), based on a recent regional survey (Sha et al., unpublished data). Detailed information on all strains used in this study is provided in Table 1. All fungal strains used in this study are maintained at the College of Modern Agriculture, Kashi University.

### 2.2. Pathogen Samples and DNA Extraction

Isolated strains were cultivated on potato dextrose agar (PDA) in a constant-temperature incubator at 25 °C for 7 days. Fungal mycelia were collected by scraping culture surfaces, then thoroughly homogenized using an automatic high-speed grinding system (JXFSPRP-48L, JingXin, Shanghai, China). Genomic DNA was extracted from the homogenate using a commercial DNA extraction kit (B518229, Sangon Biotech, Shanghai, China). Extracted DNA concentration and quality were assessed by UV-Vis spectrophotometer (UV1700, Lengguang Technology, Shanghai, China). DNA samples meeting quality standards (The A260/A280 ratio of the DNA samples ranged from 1.8 to 1.9, indicating high purity.) were immediately stored at −20 °C for subsequent use.

### 2.3. LAMP Primer Design

The ITS sequence of *A. alternata* was aligned with corresponding ITS sequences from congeners retrieved from GenBank, and from other pathogenic fungi isolated from *European plum* (sequenced by Tsingke Biotechnology Co., Ltd., Xi’an, China). Analysis identified highly conserved and specific regions suitable for primer targeting. Primer design was performed using online software PrimerExplorer V5 (https://primerexplorer.eiken.co.jp/lampv5/, accessed on 15 August 2025); all designed primers were commercially synthesized by Tsingke Biotechnology Co., Ltd. (Xi’an, China).

### 2.4. LAMP Assay Development and Optimization

The initial LAMP-Cresol Red visual assay was performed in a 25 μL reaction mixture comprising 10 μL of 2.5× Bst 4.0 Low Salt Mix (PC2610, solarbio, Beijing, China), 2.5 μL of each of 10× Red pH Dye (PC2610, solarbio, Beijing, China), 10× LAMP Primer Mix, and DNA template, and 7.5 μL of Nuclease-free water (R0581, solarbio, Beijing, China). The ratio of inner to outer primers was maintained at 8:1. The reaction mixture was incubated at 65 °C for 1 h in a constant-temperature water bath (LICHEN, Shangha, China), followed by enzyme inactivation at 80 °C for 10 min to terminate the reaction. Negative controls were prepared by replacing the DNA template with an equivalent volume of Nuclease-free water.

The LAMP-SYBR Green I real-time fluorescence assay was performed in a 25 μL system containing 10 μL of 2.5× Bst 4.0 Low Salt Mix (PC2610, solarbio, Beijing, China), 2.5 μL of each of 10× SYBR Green I (SY1020, solarbio, Beijing, China), 10× LAMP Primer Mix, and DNA template, and 7.5 μL of Nuclease-free water (R0581, solarbio, Beijing, China), with an inner-to-outer primer ratio of 8:1. Amplification was performed at 65 °C for 1 h using a real-time PCR system (Q2000B, Long Gene, Hangzhou, Zhejiang, China), with a final step of 80 °C for 10 min for enzyme denaturation. Negative controls were included by substituting the DNA template with Nuclease-free water. Based on the manufacturer’s 10,000× stock, 1× corresponds to a 10,000-fold dilution. Thus, the working range of 0.2×–2.0× represents an approximate final dye concentration on the order of 0.2–2.0 μM, and the optimal range (0.8×–1.0×) corresponds to approximately 0.8–1.0 μM in the reaction mixture.

In the real-time fluorescence assay, amplification data were recorded in cycle mode, with each cycle representing a fixed data acquisition interval of 45 s. Fluorescence amplification curves were plotted as a function of cycle number.

LAMP reaction conditions (temperature, duration, and component proportions) were systematically optimized to detect *A. alternata* using the primers. A gradient optimization of DNA template volume (1, 2, 3, 4, 5, 6, 7, 8 μL) was first performed. This was followed by a temperature gradient optimization (62, 63, 64, 65, 66, 67, 68, 69 °C) to determine the optimal amplification temperature. The reaction time was then optimized (30, 35, 40, 45, 50 min) at the identified optimal temperature. Finally, with all aforementioned parameters set to their optimal conditions, the concentration of SYBR Green I in the LAMP-SYBR Green I system was further optimized through a gradient analysis (0.2, 0.4, 0.6, 0.8, 1.0, 1.2, 1.4, 1.6, 1.8, and 2.0×). Results were preliminarily assessed by observing the color change in the visual system and the amplification curves in the real-time fluorescence detection system.

After brief centrifugation, 3 μL of each reaction product was subjected to 1.2% agarose gel electrophoresis prepared in 1× TAE buffer at 220 V and 150 mA for 12 min. Agarose gel electrophoresis was performed in a physically separated area equipped with ventilation and sterilization facilities. Each run was preceded by routine sterilization and ventilation, and the electrophoresis area was strictly separated from the amplification area. For product verification, the smallest ladder fragment was excised from the agarose gel, purified, and subjected to Sanger sequencing. The obtained sequence was analyzed using BLASTn implemented in the NCBI BLAST suite (https://blast.ncbi.nlm.nih.gov/Blast.cgi (accessed on 15 August 2025)) against the ITS sequence of *A. alternata*.

### 2.5. LAMP Assay Specificity

To determine the specificity of the LAMP assay, *A. alternata* was tested alongside other fungal pathogens. These included common pathogens found on *European plum*, and the eight aforementioned species prevalent on fruit trees in the Xinjiang region. In actual research, certain *Alternaria* strains and *Monilinia* strains were not isolated from the diseased *European plum* trees used in this study, and therefore subsequent diagnostic method development and specificity validation were focused on *A. alternata*. Both the LAMP-Cresol Red visual and LAMP-SYBR Green I real-time fluorescence assays were performed under their respective optimal parameters and reaction compositions. A negative control using Nuclease-free water instead of DNA template was included for each system.

### 2.6. LAMP Assay Sensitivity

The concentration of extracted DNA (246 μg/mL) was determined using a UV–Vis spectrophotometer (UV1700, Lengguang Technology, Shanghai, China). Genomic DNA of *A. alternata* was serially diluted ten-fold with nuclease-free water to generate a concentration gradient. Each dilution was used as a template for amplification using the optimized LAMP-Cresol Red visual detection system and the LAMP-SYBR Green I real-time fluorescence detection system under their respective optimal reaction conditions. All sensitivity assays were performed independently, and nuclease-free water was included as a no-template control in each run. Detection sensitivity was evaluated based on the lowest DNA concentration within the tested dilution series that produced a consistent positive amplification signal.

### 2.7. LAMP Assay Robustness

Multiple *A. alternata* isolates (Table 1) from different plant hosts and locations within the Kashgar region were analyzed using both LAMP-Cresol Red visual and LAMP-SYBR Green I real-time fluorescence detection systems. All reactions were performed under the established optimal conditions for each system, with Nuclease-free water serving as a negative control. Optimized protocols for LAMP-Cresol Red and LAMP-SYBR Green I assays were used to perform the amplification.

### 2.8. LAMP Assay Field Applicability

A rapid crude DNA extraction method for detecting field-collected *European plum* brown spot samples was adapted from the protocol of [13]. *European plum* fruits were collected from four counties (Payzawat, Yarkant, Yengisar, Markit) in the Kashgar region. From each location, three diseased and one healthy fruit were selected. Approximately 0.5 cm^2^ of pericarp tissue was excised from the lesion border of symptomatic fruits (experimental group) and from healthy fruits (control group) using a sterile scalpel, then placed in 2 mL microcentrifuge tubes. Samples were homogenized with grinding beads in an automatic high-speed grinder (JXFSPRP-48L, JingXin, Shanghai, China), mixed with 100 μL of 10× TE buffer (100 mM Tris-HCl, 10 mM EDTA, pH 8.0), incubated at 95 °C for 2 min and 85 °C for 1 min, flash-cooled at −20 °C for 1 min, and centrifuged at 9729× *g* for 30 s (2-16R, HENGNUO, Changsha, Hunan, China). Crude DNA extracts were analyzed using optimized LAMP-Cresol Red visual and LAMP-SYBR Green I real-time detection systems.

## 3. Results

### 3.1. LAMP Primer Design and Selection

**Figure 1:**
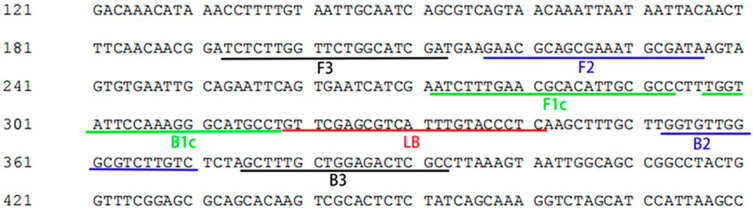
Localization of LAMP Primers 2 on the ITS Gene Sequence.

Detection sensitivity and specificity are often influenced by target gene sequence and designed primer characteristics. Specific primers were developed by comparing the ITS sequence of *A. alternata* with those of congeners (*A. arborescens*, *A. citriarbusti*, *A. gaisen*, *A. mali*, and *A. toxicogenica*). Sequence alignment was performed using reference sequences from Woudenberg et al. (2015) [29]. The primer set consisted of two outer primers (F3, B3), two inner primers (FIP, BIP), and one loop primer (LB)—the latter included to enhance reaction speed and assay sensitivity [30]. The F3 primer corresponds to the forward sequence of positions 193 to 212, and the B3 primer to the reverse complement of positions 375 to 392. The FIP primer is composed of the forward sequence of F2 (positions 217 to 236) and the reverse complement of F1c (positions 272 to 293). The BIP primer consists of the forward sequence of B1c (positions 297 to 318) and the reverse complement of B2 (positions 353 to 370). The LB primer was designed as the forward sequence of positions 320 to 341. The location and sequences of the LAMP primers are detailed in Figure 1 and Table 2, respectively.

### 3.2. LAMP Reaction System Optimization

The LAMP-Cresol Red visual system achieved optimal performance at 65 °C with a 45 min incubation time, using 3 μL of DNA template. For the LAMP-SYBR Green I real-time fluorescence detection system, the optimal reaction temperature was 66 °C with 3 μL of DNA template. Amplification curves indicated that exponential amplification initiated around 15 min for most reactions, reaching maximum fluorescence intensity by ~50 min.

Fluorescence intensity of SYBR Green I at concentrations ≤ 0.6× was barely detectable by the real-time PCR system (Q2000B, Long Gene, Zhejiang, China). At concentrations ≥ 1.2×, the instrument initially detected abnormally high non-specific fluorescence, which then dropped sharply because of inhibition of DNA polymerization by excessive SYBR Green I. While both 0.8× and 1.0× concentrations yielded similar amplification timing, the 1.0× concentration produced significantly higher fluorescence intensity. Therefore, the optimal volume of 10× SYBR Green I was determined to be 2.5 μL per reaction.

**Figure 2:**
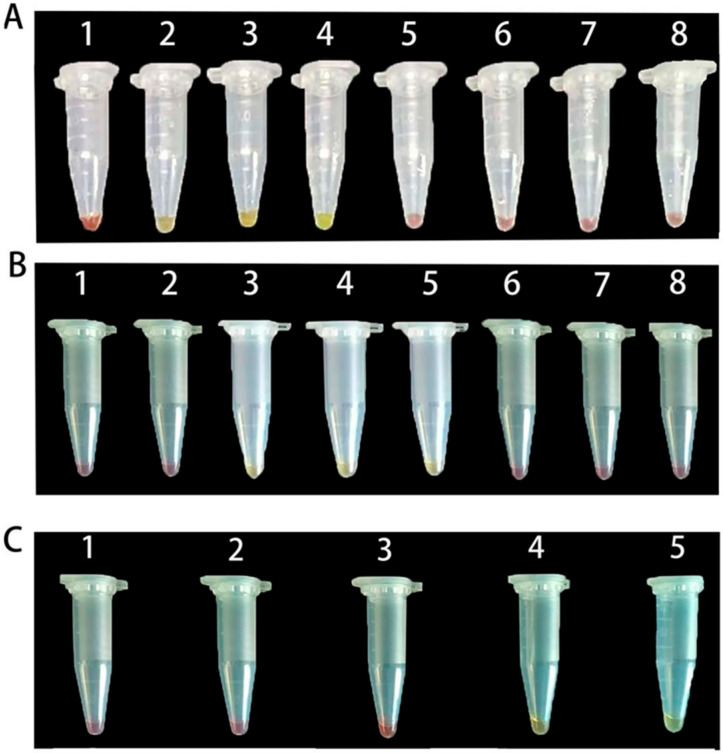
Optimization of the LAMP–Cresol Red visual detection system. (**A**): Optimization of DNA input; lanes 1–8 correspond to 1, 2, 3, 4, 5, 6, 7, and 8 μL, respectively. (**B**): Temperature optimization; lanes 1–8 correspond to 62, 63, 64, 65, 66, 67, 68, and 69 °C, respectively. (**C**): Reaction-time optimization; lanes 1–5 correspond to 30, 35, 40, 45, and 50 min, respectively.

**Figure 3:**
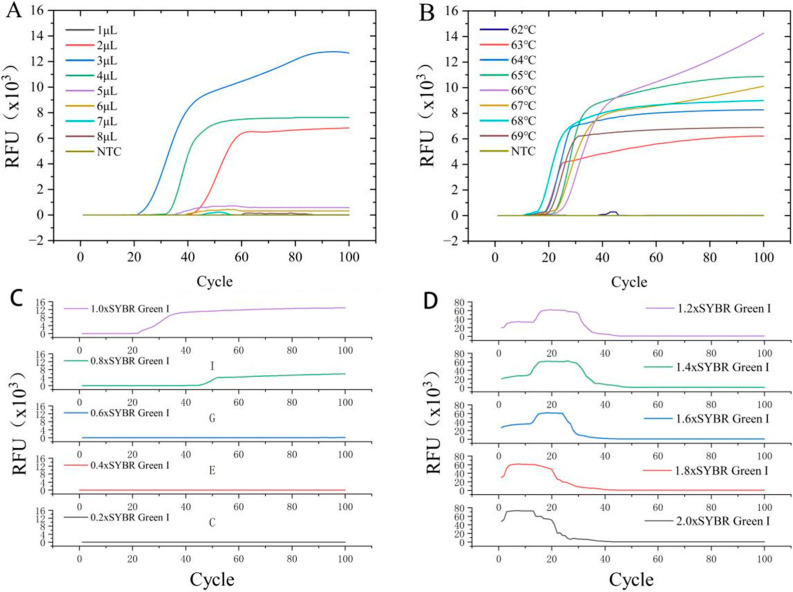
Optimization of the LAMP-SYBR Green I real-time fluorescence detection system. (**A**) Optimization of DNA input; amplification curves correspond to 1, 2, 3, 4, 5, 6, 7, 8 μL and NTC, respectively. (**B**) Temperature optimization; amplification curves correspond to 62, 63, 64, 65, 66, 67, 68, 69 °C and NTC, respectively. (**C**,**D**) Optimization of SYBR Green I concentration: panel (**C**), 0.2–1.0× SYBR Green I; panel (**D**), 1.2–2.0× SYBR Green I.

The optimization processes for the LAMP-Cresol Red visual and LAMP-SYBR Green I real-time detection systems are illustrated in Figure 2 and Figure 3, respectively. The corresponding optimized reaction compositions are detailed in Table 3 and Table 4, while the 10× LAMP Primer Mix formulation is provided in Table 5.

### 3.3. LAMP Specificity Detection

**Figure 4:**
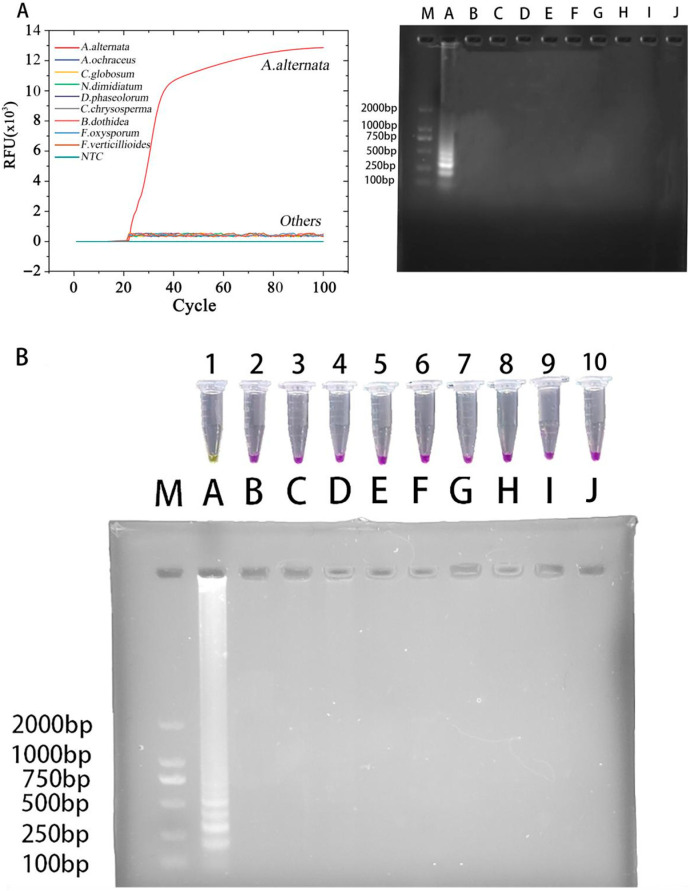
LAMP specificity assay results. (**A**) Specificity evaluation of the LAMP-SYBR Green I real-time fluorescence monitoring system. (**Left**), real-time amplification curves; (**right**), agarose-gel electrophoresis results. M: DNA 2K plus marker; lanes A–J: *A. alternata*, *A. ochraceus*, *C. globosum*, *N. dimidiatum*, *D. phaseolorum*, *C. chrysosperma*, *B. dothidea*, *F. oxysporum*, *F. verticillioides*, and NTC, respectively. (**B**) Specificity assessment of the LAMP-Cresol Red visual detection system. M: DNA 2K plus marker; tubes 1–10 (corresponding to lanes A–J): *A. alternata*, *A. ochraceus*, *C. globosum*, *N. dimidiatum*, *D. phaseolorum*, *C. chrysosperma*, *B. dothidea*, *F. oxysporum*, *F. verticillioides*, and NTC, respectively.

A distinct color change to yellow occurred in the LAMP-Cresol Red visual system. Specific amplification in the LAMP-SYBR Green I real-time system occurred exclusively when the template was *A. alternata* (no color change or specific amplification curves were observed when the template consisted of other pathogenic fungi or Nuclease-free water). Agarose gel electrophoresis (DYY-6C, LIUYI, Beijing, China) confirmed these findings, with no DNA bands detected using a gel documentation system (Tanon-1600, Tanon, Shanghai, China) in any non-target reaction (Figure 4).

### 3.4. LAMP Sensitivity Detection

**Figure 5:**
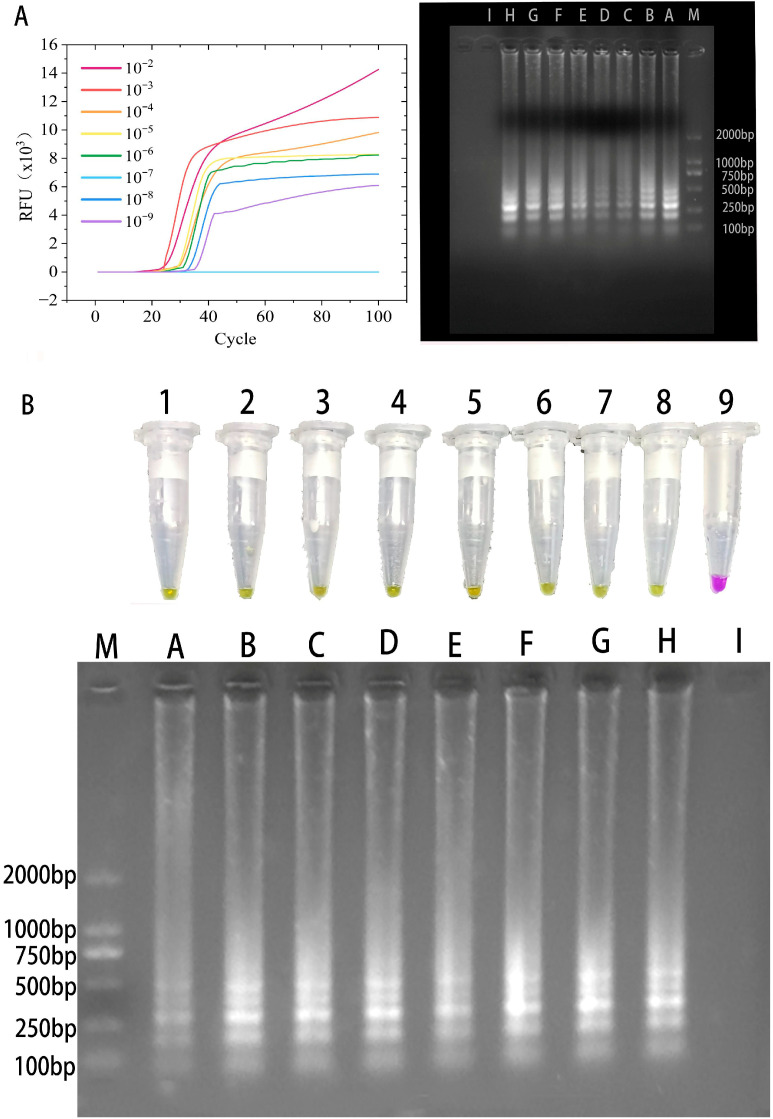
Sensitivity analysis of the LAMP assays for *A. alternata.* (**A**) Sensitivity evaluation of the LAMP-SYBR Green I real-time fluorescence detection system. (**Left**): real-time amplification curves obtained using a 10-fold serial dilution of *A. alternata* genomic DNA. (**Right**): corresponding agarose gel electrophoresis results. M: DNA 2K plus marker; lanes A–I represent 2.46 ng/μL, 0.246 ng/μL, 24.6 pg/μL, 2.46 pg/μL, 0.246 pg/μL, 24.6 fg/μL, 2.46 fg/μL, 0.246 fg/μL, and the no-template control (NTC), respectively. (**B**) Sensitivity assessment of the LAMP–Cresol Red visual detection system using the same DNA dilution series. Tubes 1–8 correspond to the same 10-fold serial dilutions shown in lanes A–H in panel (**A**), and tube 9 represents the NTC. The lowest DNA concentration tested in both systems was 0.246 fg/μL, which produced a positive amplification signal; lower concentrations were not evaluated.

Genomic DNA of *A. alternata* with an initial concentration of 246 μg/μL was serially diluted ten-fold with nuclease-free water, generating DNA concentrations ranging from 24.6 ng/μL to 0.246 fg/μL. In the LAMP-Cresol Red visual assay, a distinct color change from red to yellow was observed for all tested DNA concentrations, including the lowest tested concentration of 0.246 fg/μL. Agarose gel electrophoresis further confirmed successful amplification at each dilution by revealing the characteristic ladder-like banding pattern. Similarly, in the LAMP-SYBR Green I real-time fluorescence detection system, typical sigmoidal amplification curves were consistently observed across the same dilution range, including at 0.246 fg/μL, and were corroborated by agarose gel electrophoresis. Within the tested dilution range, positive amplification signals were consistently obtained in both LAMP detection systems down to 0.246 fg/μL. Because DNA concentrations below this level were not evaluated, this value represents the lowest tested concentration yielding a positive signal rather than a definitive limit of detection (Figure 5).

### 3.5. LAMP Robustness Test

**Figure 6:**
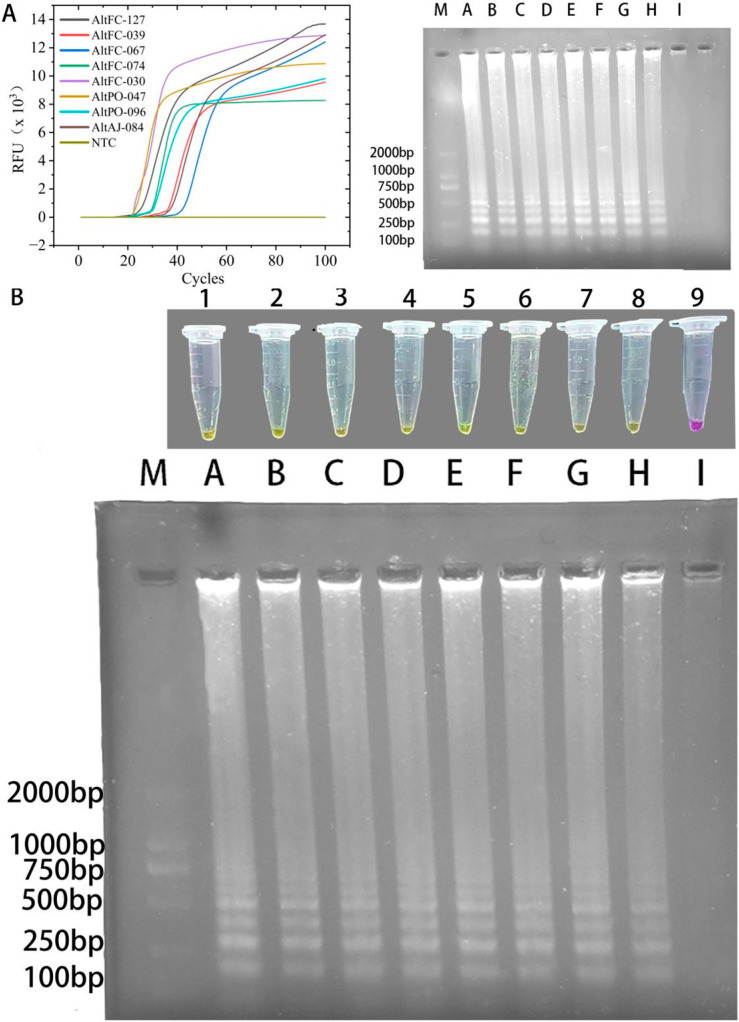
LAMP universality evaluation. (**A**) Sensitivity evaluation of the LAMP–SYBR Green I real-time fluorescence detection system. (**Left**), real-time amplification curves; (**right**), agarose-gel electrophoresis results. M: DNA 2K plus marker; lanes A–I: AltFC-127, AltFC-039, AltFC-067, AltFC-074, AltFC-030, AltPO-047, AltPO-096, AltAJ-084, and NTC, respectively. (**B**) Visual universality assessment with the LAMP–Cresol Red system. M: DNA 2K plus marker; tubes 1–9 (corresponding to lanes A–I): AltFC-127, AltFC-039, AltFC-067, AltFC-074, AltFC-030, AltPO-047, AltPO-096, AltAJ-084, and NTC, respectively.

Genomic DNA extracted from eight *A. alternata* strains isolated from different plant varieties in the Kashgar region consistently produced a positive yellow color change in the LAMP-Cresol Red visual assay. This result was confirmed by 1.2% agarose gel electrophoresis, which produced the characteristic ladder-like banding pattern indicative of successful LAMP amplification. Similarly, in the LAMP-SYBR Green I real-time detection system, all eight strains exhibited specific amplification curves; electrophoresis similarly confirmed the presence of the typical ladder pattern. In contrast, no reaction was observed in the Nuclease-free water negative control. These results demonstrate that both systems can accurately and rapidly identify *A. alternata* strains derived from diverse plant hosts and locations, and that both systems are highly robust (Figure 6).

### 3.6. LAMP Field Applicability Test

**Figure 7:**
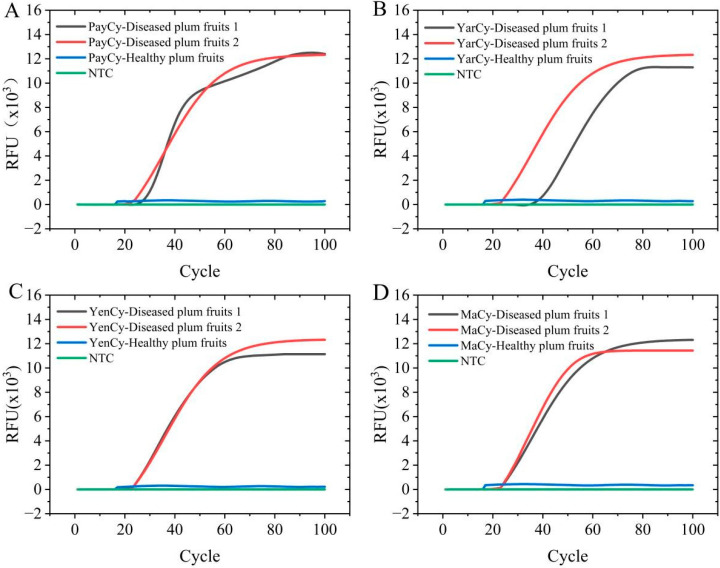
Field evaluation results of the LAMP–SYBR Green I real-time fluorescence system. (**A**) PayCy-diseased plum fruits 1, PayCy-diseased plum fruits 2, PayCy-healthy plum fruits, NTC. (**B**) YarCy-diseased plum fruits 1, YarCy-diseased plum fruits 2, YarCy-healthy plum fruits, NTC. (**C**) YenCy-diseased plum fruits 1, YenCy-diseased plum fruits 2, YenCy-healthy plum fruits, NTC. (**D**) MaCy-diseased plum fruits 1, MaCy-diseased plum fruits 2, MaCy-healthy plum fruits, NTC.

**Figure 8:**
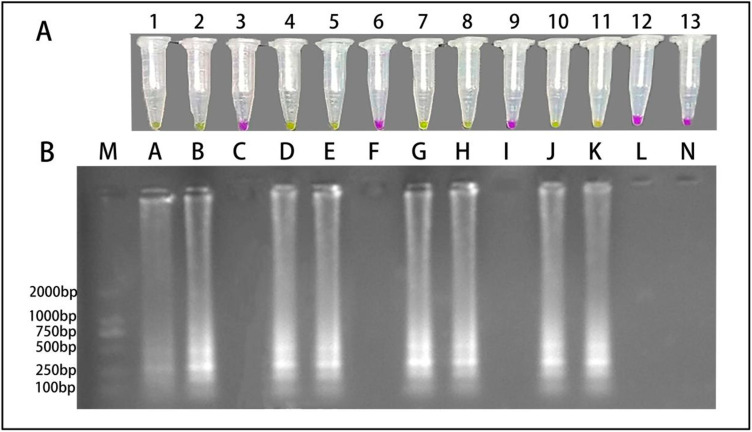
Field evaluation results of the LAMP–Cresol Red visual detection system. (**A**) Visual detection results of the LAMP–Cresol Red system. (**B**) Electrophoretic analysis on 1.2% agarose gel. M: DNA 2K plus marker; Tubes 1–13 (corresponding to lanes A–N): PayCy-diseased plum fruit 1, PayCy-diseased plum fruit 2, PayCy-healthy plum fruit, YarCy-diseased plum fruit 1, YarCy-diseased plum fruit 2, YarCy-healthy plum fruit, YenCy-diseased plum fruit 1, YenCy-diseased plum fruit 2, YenCy-healthy plum fruit, MaCy-diseased plum fruit 1, MaCy-diseased plum fruit 2, MaCy-healthy plum fruit, and NTC (lane N).

Crude DNA extracts from symptomatic fruits collected from each region induced a positive color change to yellow in the LAMP-Cresol Red visual assay. This was confirmed by 1.2% agarose gel electrophoresis, with the characteristic ladder-like amplification bands being produced. However, crude DNA extracts from healthy fruits obtained from these same regions showed no positive reaction in this system. In the LAMP-SYBR Green I real-time fluorescence detection system, crude DNA from all symptomatic fruit samples produced specific amplification curves, with electrophoresis again revealing a ladder pattern. No amplification signals were observed for any healthy control sample. Because both systems accurately and rapidly identify *A. alternata* from crude DNA extracts of infected fruits from different locations, both systems exhibit strong field applicability (Figure 7 and Figure 8).

## 4. Discussion

Accurate identification of *A. alternata* is a critical first step and fundamental basis for implementing early control strategies against *European plum* brown spot. This enables the timely application of appropriate management tactics and technical measures before symptom onset. We develop a visual LAMP assay using the colorimetric change of Cresol Red (the LAMP-Cresol Red visual system), alongside a LAMP-SYBR Green I real-time fluorescence detection system constructed with the fluorescent dye SYBR Green I. Both methods are designed for rapid and specific identification of *A. alternata*.

The variable region of the ITS gene of *A. alternata* was selected as a target, and a set of LAMP primers was designed. Because incorporating a loop primer can accelerate reaction kinetics and enhance detection sensitivity [30], we also designed a loop primer to specifically identify *A. alternata* (supplementing the standard set of four inner and outer primers). The inclusion of this loop primer contributed to more rapid and stable amplification under the optimized reaction conditions, thereby improving the overall efficiency of the LAMP assay. Although dual-loop primer systems typically enhance LAMP amplification efficiency, the high degree of conservation within the ITS region constrains the design space for species-specific loop primers. Comprehensive evaluation identified only a single loop primer candidate that fulfilled both specificity and thermodynamic stability requirements, necessitating a single-loop primer strategy in this study. To compensate for this potential efficiency deficit, amplification performance was enhanced through systematic optimization of component stoichiometry and reaction temperature, ultimately achieving detection of the target pathogen within 45 min and confirming the feasibility of this approach. It is noteworthy that during the primer design phase of this study, some primers exhibited varying degrees of homology with sequences from congeneric species, including *A. tenuissima*, *A. compacta*, *A. alstroemeria*, *A. burnsii*, *A. solani*, *A. ricini*, and *A. brassicicola*. However, a comprehensive *in silico* specificity evaluation using Primer-BLAST implemented at the NCBI website (https://www.ncbi.nlm.nih.gov/tools/primer-blast/ (accessed on 15 August 2025)) indicated that the primer set, as a whole, retained specificity. This is attributed to the fact that the forward and reverse primers were not identical across these homologous regions simultaneously. In practice, the aforementioned non-target *Alternaria* strains were not isolated from the symptomatic *European plum* trees used in this study. Consequently, they were not included in the experimental specificity validation assays. Our results demonstrate that the designed primers are feasible and specific for detecting the causal agent of brown spot on *European plum* within the Xinjiang region. Nevertheless, it must be acknowledged that they may not confer perfect discriminative power for distinguishing between all closely related species at the interspecific level within the genus *Alternaria*. This potential limitation warrants specific attention in subsequent research and validation studies.

In development of the LAMP-Cresol Red visual detection system, various strategies have been explored by researchers, leveraging the significant decrease in reaction pH resulting from the release of hydrogen ions during LAMP amplification. Xie et al. (2014) proposed a portable pH meter-based method for direct pH monitoring [31], and Zhang et al. (2014) constructed an electrochemical LAMP detection platform [32]. However, because these methods are highly dependent on specialized instruments, their applicability in field settings is limited. With technological advancements, pH indicator-based visual LAMP methods have been explored because of their operational simplicity and intuitive results. For instance, in 2015, Tanner et al. first proposed the use of pH indicator dyes for the visual display of LAMP results [33]. Skenndri et al. (2025) achieved detection of *Salmonella* spp. as low as 0.39 CFU/μL using Cresol Red [34], and Park et al. (2023) successfully detected 1 fg/μL of MKF1 plasmid using a phenolphthalein test strip method [27]. Building on prior research, we systematically optimized the reaction system and conditions. Under the optimized reaction parameters, the LAMP-Cresol Red assay enabled reliable detection of *A. alternata* genomic DNA at femtogram-level concentrations within the tested dilution range. It should also be noted that the optimal reaction temperature and incubation time established in this study were determined using standard 1.5 mL microcentrifuge tubes. These parameters may be subject to variation depending on the physical specifications (e.g., volume, wall thickness) and material composition (e.g., polypropylene) of the reaction vessels used. Therefore, empirical optimization of the reaction conditions is recommended when adapting this assay to different experimental setups or field-deployable formats to ensure consistent and maximal detection efficiency.

During development of the LAMP-SYBR Green I real-time fluorescence detection system, EvaGreen dye has been more widely used than SYBR Green I (because the latter may inhibit DNA polymerase activity at high concentrations) [35]. Consequently, this dye is typically added only after reaction completion by either opening the tube [36,37] or placing it on the tube cap to be centrifuged into the mixture post-amplification [38,39] where it serves solely for end-point fluorescence detection. We systematically evaluated the impact of SYBR Green I concentration on LAMP amplification efficiency. We demonstrate that stable and specific amplification was achieved within a dye concentration range of 0.8× to 1.0×, whereas concentrations <0.6× or >1.2× significantly compromised reaction specificity and prevented effective target amplification. These findings establish that SYBR Green I concentration affects the efficiency and specificity of LAMP reactions, and its optimization is needed to establish a reliable real-time fluorescence detection system.

Liu et al. (2022) developed a PCR-based technique to detect *A. alternata* during apple pathogen identification, achieving a detection sensitivity of 1 ng [40], and Yang et al. (2019) established a LAMP assay for *A. alternata* in pear pathogens with a sensitivity of 1 pg [41]. Molecular detection methods have also been developed for other *Alternaria* species—e.g., *Alternaria solani* [42]. Among these detection approaches, LAMP-based assays generally exhibit higher analytical sensitivity. In the present study, both detection systems consistently produced positive amplification signals at femtogram-level DNA concentrations within the tested dilution range, and specifically detected *A. alternata* without non-specific amplification of other coexisting fungal species associated with *European plum*, confirming high assay specificity.

Compared with previously reported LAMP assays for *A. alternata*, the assays developed in this study show improved analytical sensitivity, host-specific applicability to *European plum*, and complementary detection formats suitable for both field-based screening and laboratory validation.

In field testing, both the LAMP-Cresol Red visual and LAMP-SYBR Green I real-time fluorescence detection systems were highly accurate and produced consistent results. The LAMP–Cresol Red visual system can be performed using a simple isothermal heating block without complex instrumentation, whereas the LAMP–SYBR Green I real-time system enables real-time monitoring of amplification through fluorescence curves and is therefore more suitable for laboratory-based confirmation. Together, these two approaches constitute complementary detection formats supporting different diagnostic application scenarios.

It should be noted that the present study primarily focused on the development and analytical validation of the detection methods under controlled experimental conditions. The LAMP method established in this study provides a reliable tool for the rapid field detection of *A. alternata* on plum fruits. Our deliberate focus on fruit tissue as the primary detection target is based on the following rationale: the fruit is the ultimate site where the disease causes direct economic loss. Consequently, validating the sensitivity and specificity of the detection method on this target tissue constitutes the most critical step in evaluating its core field-applicable value. This study demonstrates stable detection of *A. alternata* in symptomatic fruit samples, supporting its potential use for field diagnosis and disease monitoring.

It is important to acknowledge that field disease epidemiology is a continuous dynamic process. *A. alternata*, as a pathogen capable of infecting multiple tissues, has a complete infection cycle encompassing twigs, young leaves, and finally fruits. From an epidemiological perspective, overwintering of the pathogen in diseased twig tissues may serve as a primary inoculum source for the subsequent growing season. Accordingly, extension of the detection strategy to additional host tissues represents a logical direction for further investigation.

Furthermore, application of the assay to young leaf tissues may allow detection of low pathogen concentrations prior to the appearance of visible symptoms, given the high sensitivity of LAMP-based amplification. Such applications represent a potential direction for future research, but require further validation.

Overall, these results indicate that the developed LAMP assays provide a methodological basis for detecting *A. alternata* across different stages of infection. Future studies will be necessary to evaluate assay performance in additional tissue types and to assess its utility within broader disease monitoring frameworks.

## 5. Conclusions

This study presents the development of two novel, highly sensitive methods for the early detection of *A. alternata*, the pathogen responsible for brown spot disease in *European plum* (*Prunus domestica* L.), a major global crop. Through the integration of LAMP-based technologies, both LAMP-Cresol Red chromogenic and LAMP-SYBR Green I real-time fluorescent detection methods exhibit remarkable specificity and sensitivity, enabling reliable detection of *A. alternata* genomic DNA at femtogram-level concentrations within the evaluated range. These LAMP-based assays provide substantially enhanced analytical performance compared with conventional PCR-based techniques while offering simpler operational requirements. Our findings not only enhance the capacity for early disease detection in plum production but also offer a promising tool for rapid diagnosis and disease monitoring. This research lays the groundwork for the sustainable development of the *European plum* industry, particularly in China, and contributes to the broader goals of agricultural biosecurity and crop protection on a global scale. The application of these methodologies may facilitate early disease surveillance and support informed management decisions in *European plum* production systems.

## Figures and Tables

**Table 1:** Fungal strains (all from Kashi, Xinjiang, China) used for molecular analysis in this study.

Isolates	Species	Host	SYBR Green I ^a^	Cresol Red ^a^	GenBank Accession Numbers
AltPD-017	*Alternaria alternata*	*Prunus domestica*	+	+	PX122071.1
AltPD-042	*A. alternata*	*Prunus domestica*	+	+	PX122072.1
AltPD-069	*A. alternata*	*Prunus domestica*	+	+	PX122073.1
AltPD-091	*A. alternata*	*Prunus domestica*	+	+	PX229955.1
AltPD-105	*A. alternata*	*Prunus domestica*	+	+	PX229956.1
AspPD-127	*Aspergillus ochraceus*	*Prunus domestica*	−	−	PX247016.1
ChaPD-153	*Chaetomium globosum*	*Prunus domestica*	−	−	PX247017.1
NeoPD-176	*Neoscytalidium dimidiatum*	*Prunus domestica*	−	−	PX251636.1
NigPD-198	*Nigrospora* sp.	*Prunus domestica*	−	−	PX247018.1
AltFC-127	*A. alternata*	*Ficus carica*	+	+	PX106383.1
AltFC-039	*A. alternata*	*Ficus carica*	+	+	PX106374.1
AltFC-067	*A. alternata*	*Ficus carica*	+	+	PX108872.1
AltFC-074	*A. alternata*	*Ficus carica*	+	+	PX106380.1
AltFC-030	*A. alternata*	*Ficus carica*	+	+	PX111643.1
AspFC-039	*A. ochraceus*	*Ficus carica*	−	−	PX225979.1
FusFC-027	*Fusarium oxysporum*	*Ficus carica*	−	−	PX225984.1
FusFC-013	*Fusarium verticillioides*	*Ficus carica*	−	−	PX225983.1
FunFC-021	*Fungal endophyte*	*Ficus carica*	−	−	PX225982.1
FunFC-020	*F. endophyte*	*Ficus carica*	−	−	PX225981.1
CytFC-005	*Cytospora chrysosperma*	*Ficus carica*	−	−	PX225980.1
NeoFC-013	*N. dimidiatum*	*Ficus carica*	−	−	PX225986.1
NeoFC-030	*N. dimidiatum*	*Ficus carica*	−	−	PX225985.1
AltPO-047	*A. alternata*	*Platanus orientalis*	+	+	PX121640.1
AltPO-096	*A. alternata*	*Platanus orientalis*	+	+	PX121642.1
CytPO-092	*C. chrysosperma*	*Platanus orientalis*	−	−	PX225989.1
CytPO-019	*C. chrysosperma*	*Platanus orientalis*	−	−	PX225987.1
CytPO-081	*C. chrysosperma*	*Platanus orientalis*	−	−	PX225988.1
DiaPO-022	*Diaporthe eres*	*Platanus orientalis*	−	−	PX225990.1
LasPO-046	*Lasiodiplodia* sp.	*Platanus orientalis*	−	−	PX225991.1
LasPO-153	*L.* sp.	*Platanus orientalis*	−	−	PX225992.1
AltAJ-084	*A. alternata*	*Albizia julibrissin*	+	+	PX106169.1
BotAJ-065	*Botryosphaeria dothidea*	*Albizia julibrissin*	−	−	PX238467.1
BotAJ-029	*B. dothidea*	*Albizia julibrissin*	−	−	PX238465.1
BotAJ-072	*B. dothidea*	*Albizia julibrissin*	−	−	PX238468.1
BotAJ-041	*B. dothidea*	*Albizia julibrissin*	−	−	PX238466.1
DidAJ-090	*Didymella* sp.	*Albizia julibrissin*	−	−	PX238469.1
NeoAJ-025	*N. dimidiatum*	*Albizia julibrissin*	−	−	PX238470.1
SchAJ-034	*Schizophyllum commune*	*Albizia julibrissin*	−	−	PX238471.1
SchAJ-062	*S. commune*	*Albizia julibrissin*	−	−	PX238473.1
SchAJ-037	*S. commune*	*Albizia julibrissin*	−	−	PX238472.1

^a^ +: positive reaction; −: negative reaction.

**Table 2:** Loop-mediated isothermal amplification (LAMP) primers targeting the ITS gene used in this study.

Primer	Length	Sequence(5′-3′)
AltPD-F3	20	TCTCTTGGTTCTGGCATCGA
AltPD-B3	18	GCGAGTCTCCAGCAAAGC
AltPD-FIP	42	GGCGCAATGTGCGTTCAAAGAT GAACGCAGCGAAATGCGATA
AltPD-BIP	40	TGGTATTCCAAAGGGCATGCCT GACAAGACGCCCAACACC
AltPD-LB	22	GTTCGAGCGTCATTTGTACCCTC
AltPD-F1c	22	GGCGCAATGTGCGTTCAAAGAT
AltPD-B1c	22	TGGTATTCCAAAGGGCATGCCT
AltPD-F2	20	GAACGCAGCGAAATGCGATA
AltPD-B2	18	GACAAGACGCCCAACACC

**Table 3:** LAMP-Cresol Red Visual Assay.

Serial Number	Reaction Reagent	Concentration/Specification	Added Volume (μL)
1	Bst4.0LowSaltMix	2.5×	10
2	Red pH Dye	10×	2.5
3	LAMP Primer Mix	10×	2.5
4	DNA	—	3
5	ddH_2_O	—	7
	Total	—	25

**Table 4:** LAMP-SYBR Green I real-time fluorescence quantitative detection system.

Serial Number	Reaction Reagent	Concentration/Specification	Added Volume (μL)
1	Bst4.0LowSaltMix	2.5×	10
2	SYBR Green I	10×	2.5
3	LAMP Primer Mix	10×	2.5
4	DNA	—	3
5	ddH_2_O	—	7
	Total	—	25

**Table 5:** 10× LAMP primer master mix.

Serial Number	Reaction Reagent	Concentration/Specification	Added Volume (μL)
1	FIP	100 μM	16
2	BIP	100 μM	16
3	F3	100 μM	2
4	B3	100 μM	2
5	LB	100 μM	4
6	ddH_2_O	—	56
	Total	—	100

## Data Availability

The original contributions presented in this study are included in the article. Further inquiries can be directed to the corresponding author.
